# More food for thought: a follow-up qualitative study on experiences of food bank access and food insecurity in Ottawa, Canada

**DOI:** 10.1186/s12889-022-13015-0

**Published:** 2022-03-25

**Authors:** Anita Rizvi, Aganeta Enns, Lucas Gergyek, Elizabeth Kristjansson

**Affiliations:** School of Psychology, Faculty of Social Sciences, University of Ottawa, 136 Jean-Jacques-Lussier Pvt, Room VNR5015, Ottawa, ON K1N 6N5 Canada

**Keywords:** Food banks, Food insecurity, Physical health, Mental health, Longitudinal study, Qualitative research, Canada

## Abstract

**Background:**

Despite the widespread proliferation of food banks in high-income countries over the past several decades, there is a paucity of data regarding the long-term experiences of the people who rely on food banks. We were unable to find any other studies with follow-up interviews later than 6 months after baseline.

**Objective:**

This study examined the changes in the lived experiences of people who accessed food banks over a period of 18 months.

**Methods:**

Semi-structured interviews were conducted with 11 people who accessed food banks in Ottawa, Canada and who had participated in a 6-month study that ended one full year before this follow-up study was done. Transcripts of the interviews were analyzed through a general inductive approach involving repeated readings and coding of relevant segments of text with NVivo software according to themes that emerged iteratively. Code reports were then used to discuss and reach consensus on a final set of themes.

**Results:**

Three main themes emerged: (1) chronic physical and mental health issues intersecting with food bank access; (2) psychosocial impact of relying on food banks; and (3) living on a low income and dealing with poverty. Chronic physical and mental health conditions were prevalent among the participants. As well, 10 of the 11 participants in this 18-month follow-up continued to rely on food banks as a regular resource – not as an emergency relief measure – to supplement their nutritional needs. While most of the participants reported that food banks helped them in some way, many shortcomings were also noted regarding food amounts, quality and choice. Overall, there was little change reported since the 6-month follow-up.

**Conclusions:**

The shortcomings reported by participants can mostly be attributed to the dependence of food banks on charitable donations; thus, despite the commendable work of food bank staff and volunteers, participants described the food assistance as inadequate. Additionally, long-term food bank usage was a common denominator in the lived experiences of all our participants; therefore, our findings reinforce the need for assistance programs that target long-term food insecurity and its underlying causes, to replace or supplement charity-based food bank programs.

**Supplementary Information:**

The online version contains supplementary material available at 10.1186/s12889-022-13015-0.

## Introduction

According to the UN Universal Declaration of Human Rights, all human beings have the right to adequate food to ensure a standard of living required for the health and well-being of a person and his or her family [1], and yet 12% of households in Canada report some level of food insecurity [2]. Household food insecurity is defined as the inadequate or insecure access to food due to financial constraints [2]. It is an urgent problem in Canada that adversely impacts mental, physical, and social health, and significantly affects our health care system [2–4].

Drawing on data for 103,500 households from Statistics Canada’s Community Health Survey conducted in 2017 and 2018, researchers estimated that there were 4.4 million people, including more than 1.2 million children under the age of 18, living in food-insecure households. These results are higher than any previous national estimate, suggesting that the issue of food insecurity is a growing and pervasive public health problem in Canada [2].

Several studies have found that adults living in food-insecure households report poorer physical health and are more at risk of experiencing a wide range of chronic conditions such as heart disease, diabetes, hypertension, and arthritis [3, 5–7]. A recent literature review [3] describes six studies that found an especially strong relationship between food insecurity and poor mental health, with the risk of experiencing depression and anxiety disorder increasing with the severity of food insecurity. The growing problem of food insecurity has largely fallen on charitable organizations such as food banks to deal with [8]. This reality is reflected in the annual report of the Ottawa Food Bank organization [9], which shows that 2.2% of its revenue came from government funding, while 97.8% came from private sector and individual donations.

Sociologist Janet Poppendieck has suggested that charity food relief programs are incapable of handling the rising hunger in their communities in an effective way, and has attributed the failure of food charity programs in managing rising hunger to the unpredictability that is inherent in a system reliant on charitable donations [10]. As such, the amount of food is not sufficient, does not always meet the recipient’s preferences, dietary or otherwise, and the nutritional adequacy of the food provided may be questionable. According to Poppendieck, these shortcomings, combined with the charitable nature of the food bank system, all take a toll on human dignity through the constructed identities of “haves” and “have-nots.” Canadian food insecurity experts Riches and Tarasuk also wrote that: “Canada’s entrenched system of food charity has proven itself to be an ineffective response to household food insecurity and should be understood as part of the problem not the solution to domestic food insecurity” [11] (p. 56).

In several studies of food bank access, there were reports of food received being spoiled, harmful, or unfitting for a person’s dietary requirements [12–14]. Even though past research has shed light on the limitations of food banks in being able to provide adequate, nutritious, and diet-specific foods for the food-insecure, the number of visits to food banks in Canada rose by 28% between 2008 and 2016 [15].

Despite the proliferation of food banks in Canada, little is known about the long-term lived experiences of people who visit these agencies. An extensive 25-year-long study examining program data from a large food bank organization in Vancouver, Canada, found that the majority of people came for relatively few visits, but that 9% engaged in longer-term or ongoing usage over several years, accounting for 65% of all visits [16]. Other studies have also found that the majority of visits to food banks are by people who rely on them on a long-term basis [17, 18]. In spite of this, we did not find any previous research that follows up on people’s experiences with accessing food banks, conducted more than six months after the initial interview. Paralleling the result of our background search, a scoping review by Middleton et al. [19] analysed 286 articles on food bank access in high-income countries and found only 20 articles that included interviews with food bank clients. Of these 20 articles, only 2 described follow-up interviews, all of which were conducted within 6 weeks of the original interview.

To contribute new evidence to this area of research, our 18-month follow-up study examines the long-term experiences of food insecurity and food bank access of 11 participants who were part of the cohort in a 6-month study that was completed one year earlier by our team [20].

## Methods

### Participants and setting

The participants in this study were people who accessed any one of eleven food banks in Ottawa, Ontario, Canada in 2019. All of the participants had been interviewed in a previous phase of the study [20], completed in 2018, which examined the experiences of food bank access at two time points: baseline and 6 months. As such, the participants in the current study were familiar with the reasons for doing the research.

There were 29 participants at the original baseline time point and 20 at the 6-month follow-up (i.e., nine lost to attrition at that time). Of the 20 participants that completed the 6-month interviews, we were able to reach 11 whom we invited to participate in the 18-month follow-up. We were unable to contact the missing participants, by phone or email, despite several attempts.

The sample at our 18-month follow-up was comprised of five female and six male participants who ranged from 25 to 68 years of age, with a mean age of 49. In terms of primary source of income, two participants were receiving old age pensions, one was employed, three were receiving government disability benefits, and five were receiving social assistance benefits.

We conducted the interviews at the baseline, 6-month and 18-month time points in tandem with a quantitative survey-based study of the same eleven food banks in Ottawa, which examined the long-term impacts of various food banking approaches on food insecurity [21, 22]. Approximately one in ten of the participants in the quantitative study (*n* = 401) were invited, after completing the baseline survey, to participate in the interviews for this study. Eligibility was not limited by how long the participants had accessed a food bank when they were recruited, so our sample in the current follow-up included people who had accessed a food bank for any length of time of at least 18 months.

### Data collection

Data collection involved semi-structured interviews conducted with the 11 participants. By referring to the interview guide (see Additional file 2) which had been used in the 6-month follow-up [20], our interviewers asked the participants to share their experiences; as such, the interview guide provided a ‘rough framework’ which the interviewers used to begin each segment of the interview. The interview guide included questions on whether participants continued to access a food bank or not, changes in life circumstances related to food insecurity and food bank access, as well as changes in the general experiences and interactions at the food bank.

All participants had signed a consent form approved by the Research Ethics Board of the University of Ottawa during the previous 6-month study and were informed at the start of each interview that they could withdraw at any time without consequence. The interviews were conducted over the phone and from a private office by a trained interviewer (AR, female) or by a student (LG, male; two interviews under the direct supervision of AR). The lead investigator of the 6-month follow-up (AE) also instructed AR on specific considerations for interviewing people who access food banks. The average interview duration was approximately 30 min. The interviews were audio-recorded with the participants’ permission and the interviews were later transcribed verbatim.

Because the number of participants in qualitative studies tends to be small, attrition can be a significant problem for follow-up studies [23]. The risk of attrition is even greater in studies with populations that are considered to be marginalized [24]. By integrating strategies from previous longitudinal studies [24, 25], along with feedback from community food bank partners in Ottawa, we applied three means for increasing our retention rate. Firstly, the research purpose was relevant to participants and was clearly communicated. Secondly, we provided an incentive to participants in the form of a $10 grocery gift card for each interview they took part in. Participants were assured they would still receive the incentive, even if they chose to discontinue the interview. Lastly, we offered interview times that were convenient for the participants, including evenings and weekends.

### Data analysis

Interviews were transcribed by the paid online service Rev.com, and then the transcript files we received were compared with the audio recordings for accuracy. Thematic analysis of the interview transcripts was conducted using the general inductive approach, as outlined by Braun and Clarke [26] and Thomas [27]. We used the coding grid developed in the 6-month follow up as a starting point and revised it to reflect categories on changes regarding food bank use, quality of food provided, perceived health, etc., as reported by the participants in the interview transcripts. The interviews from each of the eleven participants were independently read by two members of our research team (AR, LG) and then double coded using NVivo software (https://www.qsrinternational.com). After repeated discussions and examinations of the independent coding reports, we reached consensus on a final set of themes. Verbatim quotes from the interviews are presented in the Results section below to elucidate the themes.

### Reporting

We used the Consolidated Criteria for Reporting Qualitative Research (COREQ) checklist [28] to improve the reporting of this study (Additional file 1).

## Results

The participants answered questions on whether their experiences and personal circumstances had changed in the previous six months. Many of the participants reported various changes which were not related to food insecurity or accessing food banks, such as quitting smoking, taking more daily walks, friends moving away, using Facebook more, breaking up with a life partner, and being displaced because of a fire in their building. For questions on changes in experiences with food banks, most of the participants’ responses were that there had been little or no change.

The following are the three main themes that emerged on analyzing our data: (1) chronic physical and mental health issues intersecting with food bank access, (2) psychosocial impact of relying on food banks, and (3) living on a low income and dealing with poverty. We elaborate on the three themes below, using excerpts from the interview transcripts to elucidate each theme.

### Chronic physical and mental health issues intersecting with food bank access

The participants predominantly reported coping with chronic health issues, both mental and physical, in conjunction with varied experiences regarding their access to food banks. The excerpt below describes the experiences of a single mother in her late twenties coping with such difficulties and, in particular, a progression from compromised physical health to episodes of depression and panic attacks:

*“I've been dealing with a lot of fibro(myalgia) pain and more headaches and more digestive issues and pain in my foot and more tiredness… I'm really tired all the time. I have low iron, low vitamin D as well… In the middle of June, like June 14th, 15th, I saw my doctor for mental health. For depression and anxiety. I had an emotional… I got really, what's it called? I was very depressed and I had panic attacks... But once I realized I was in a really, really dark place because it wasn't just one thing that was going on in my life.”*

She goes on to describe how her health issues affected her food bank visits, and the adverse effect on her eating behaviour:

*“I couldn't go to the food bank because I was really sick and then I didn't want anybody to come over to my house because I was very contagious... I guess that's a change, just eating less. I guess not taking care of myself as eating wise as I should. Does that make sense? I don't know.”*

(Participant # 10, female).

Another participant, a middle-aged woman with diabetes, described the lack of foods at the food bank for accommodating special diets:

*“My diet is the same because I’ve been diabetic, for like two years. They need to provide more things to the people who have special needs and special diets, which they don’t... I've tried to talk to them, (about accommodating a specialized diet for diabetes) and they said, no, what they get is what they get. When I left there, I had peanut butter, they had no brown bread, so I had some peanut butter, I have a pound of beef, and they gave me three cans of lentils. They had no brown rice, I got a cantaloupe, and I think that was just about it. Like I walk over there, I'm like, "Jesus, I wish that I wouldn't have gone."*

(Participant # 8, female)

The following participant, a woman in her late thirties, also described compounded health problems and expressed the difficulty of managing her health conditions with an income that was insufficient to meet her needs:

“I don’t have an employment. Like I get ODSP (Ontario Disability Support Program). Because I have a heart condition too. First, I have a heart condition, oh boy. But they fixed that. But then I have another heart condition then they fixed that again! And then two years later I became diabetic, I’m like, holy s**t, how much can one person take?... Oh my god, just now for this month I had to spend $140 to buy test strips to check my sugar because ODSP only covers 100 tests a year and if you have to test your blood sugar once a day, 100 tests only covers three months. I’m supposed to test my blood once a day but normally I don’t.”

*(Participant # 1, female).*

A middle-aged male participant shared coping with a chronic heart health issue:

*“…that’s something that's been ongoing for over a year…it's called left ventricle hypertrophy. It's a swollen ventricle due to chronic high blood pressure.”*

(Participant # 3, male)

Other participants also reported dealing with chronic physical and mental health concerns:

*“…yeah. I have osteoarthritis.”*

(Participant # 9, female).

*“I have an ongoing issue with an eating disorder. When things get stressful, I tend to stop eating. I restrict my food intake. I weigh myself constantly. I weigh my food. I just get really bizarre about it.”*

(Participant # 5, male).

*“My mental health has actually been very good relative to even probably even within six months because I've been on a new medication which has helped a lot.”*

(Participant # 4, female).

One participant described needing supplemental nutrition drinks (“Ensure”) for sustenance due to a chronic health concern (it is difficult for food banks to offer specialty items like Ensure, which are also expensive to buy):

*“I use the food bank just to get coffee or stuff like that. I haven't eaten solid food in over a year because of Crohn’s. I’ve had it since I was a kid…it’s just flared up; it’s flared up a lot…all I live on now is Ensure”.*

(Participant # 7, male).

#### Psychosocial impact of relying on food banks

This theme relates to the psychosocial aspect of asking for and receiving food assistance from a food bank. Several of the participants’ accounts conveyed a tone of resignation wherein reliance on a food bank means accepting a ‘second-class-citizen’ status, as when describing being offered the food that other people do not want. The following excerpt illustrates one participant’s experience with regard to the food received:

*“Basically they can't sell it anymore in the stores because it's all organic stuff. It will only last like a week anyway, not even. We get whatever they can't sell.”*

(Participant # 2, male).

There were frequent reports of frustration due to inconsistencies in dealing with the food bank. The following extract describes the perspective of a woman in her late thirties:

*“Frustrating… they say that after your first time you don't have to bring proof of address but then they request proof of address every time apparently. I actually wasn't able to get food last month for that reason ... I don't even bother going on Fridays since they're only open in the morning. It's a zoo and they can be very difficult too.”*

(Participant # 4, female).

Another participant reported a similar experience of frustration combined with the uncertainty of food being available when arriving at the food bank. This quote and those above from other participants convey a sense of degradation, of being part of a marginalized social group that is competing for handouts of food:

*“Because when I went, it seem it was like you have to get there early Tuesday morning to get anything at all. If you go Wednesday, Thursday, it's like it's not even worth going. And when you go, it’s just like a line up of 40 people or something. By the time they get around to you, oh, everything is gone.”*

(Participant # 1, female).

#### Living on a low income and dealing with poverty

While improvements in the food offered at the food bank were noted by a few participants, the majority continued to express the difficulties and challenges associated with trying to stretch their budget to sustain themselves day to day:

*“Well, yes. Compared to last year, this year, I must admit they did have a little bit more fruits and vegetables. Last year, I would go and sometimes, they would have no fruits and vegetables. It will be like maybe one orange and one apple, that's it. This year, they do have a little bit more fruits and vegetables... So, I must give them a little bit of credit that way. “*

(Participant # 1, female).

The same participant went on to describe the struggle to stretch the food they receive while living on a low income:

*“They don't give out a lot though, that's the problem. What they give you, it's not enough to even make a good meal. You have to mix it with other things to try to do it, and the problem is, you only can do it twice a month. So, if you're short, it's not much.”*

(Participant # 1, female).

The participant above was the only one of the eleven to report visiting a food bank more than once per month, which some food banks in Ottawa allow. Typically, the frequency of visits is limited to once per month, with the aim of providing about three days worth of food for a household.

*“You're only allowed once a month, so that's all I can go is once a month.*

*I've noticed… they had whole chickens, and they were only reserving those for families, and I was a little disappointed in that. … I have to go out and have somebody live with me just so I can get a little bit of extra food.”*

(Participant # 11, single male).

The following participant referred to the food bank as an emergency resource in her answer to how often she had accessed a food bank in the previous six months:

*“Well it's an emergency only food bank, I can only go once a month.”*

(Participant # 4, female).

Another participant described her financial hardships, which were aggravated due to illness:

*“I was too sick to go (to the food bank). But when I didn't go, I really drained my bank account more on groceries... So normally when I do our grocery, it costs like $150 and for someone who gets only like, $1,400 estimating a month, if you go and do that every two weeks, it really adds up. So when I didn't go to the food bank it really was financially straining.*”

(Participant # 10, female).

One older adult had stopped accessing the food bank because he had started to receive his pension:

*“I probably haven't been [to the food bank] I'm trying to remember if I've been there in the last six months. March is when my pension started. Yeah, I used to go regularly, monthly.”*

(Participant # 6, male).

## Discussion

In Canada, nearly equal proportions of men and women draw on food banks [16]. Our sample of participants was similar in this regard, with 5 females and 6 males taking part in our follow-up interviews.

Our findings corroborated those of other studies, that reliance on food banks as a long-term resource to help meet one’s basic needs was routine for our participants across different food banks and over time. Food banks were created as emergency short-term responses aimed at helping people cope with financial challenges such as temporary job lay-offs. However, they are habitually utilized as long-term resources by individuals with inadequate income to meet their fundamental household needs [16]. One of the participants (#4) in our study voiced this contradiction during her interview: “it's an emergency only food bank, I can only go once a month” – even though, like the other participants, she had relied on the food bank since the start of the 18-month study.

The prevalence of long-term food bank access by the interview participants was also consistent with the results of our parallel quantitative study, which found that 63.8% of the 271 participants at the 18-month endpoint reported either moderate or severe food insecurity, compared to 73.0% of the 401 participants at baseline [22]. The serious extent of food insecurity after 18 months of receiving food assistance helps to understand the need to continue to rely on food banks, as was the case for most of our interview participants.

Similarities as well as differences can be noted in comparing the current 18-month follow-up study to the previous 6-month follow-up. For example, at the 6-month time point, participants reported the associated stress of living with a low-income and not being able to afford a cup of coffee, bus fare, and struggling to cope with restricted social/recreational activities due to inadequate income, which was corroborated in our findings at the 18-month time point. We found evidence of participants subsisting with the hardships linked to a limited income, especially when there were children in the home. There were several instances reported of mothers forfeiting meals or struggling to stretch the food received from the food bank to make it go further. This is consistent with other findings [29] that show that mothers in food-insecure households often forgo their meals so that their children can have more.

In addition to the stress of financial constraints, several of the participants described negative psychosocial impacts of having to rely on food banks, which people in food secure populations would not encounter. Frustration was often reported, resulting from long wait times, inconsistent proof-of-identity requirements, unavailability of diet-specific food items, and food running out before the participant’s turn came up. Our previous findings at the 6-month time point described instances of people acquiring food that either did not meet their dietary needs or had passed the best-before date [20]. Our results at the 18-month follow-up supported this finding with participant accounts of receiving food that was close to the best-before date which had to be consumed the same day. Perhaps even more serious were the participants’ perceptions that they received unwanted food – and even to get the unwanted food, the recipients had to compete by showing up at the food bank early. Van der Horst et al. noted that the “compulsory gratitude” can also feel degrading, even when recipients are able to obtain food they need [13]. Thus, despite all the valuable and commendable work that food banks accomplish, the people who need to rely on them over the long-term may come to feel that they belong to a social class that is less deserving than the general population.

Paralleling the results of the 6-month time point of our previous study, in which 19 out of 20 participants were continuing to rely on food banks to augment their limited income, our current results found 10 out of 11 participants in a similar situation. One difference noteworthy in our results at the 18-month time point was a slight improvement in the amounts of fruits and vegetables offered at some food banks, although the frustrations associated with long line-ups and protracted wait times were reported at both six months and eighteen months.

Since prior research on the lived experiences of regular and long-term use of food banks is scarce, our findings contribute new evidence attesting to the struggles that people face in trying to augment their food supply on a limited budget. A 25-year study conducted in Vancouver, Canada [16] did examine demographic and physical health factors that were correlated with extended food bank reliance, but that study did not look at the lived experiences of the food bank clients. One could describe the Vancouver study as a look “from the outside-in” whereas our study gives a long-term perspective on how food banks work, as seen through the eyes of the people who access food banks. The participants’ unique personal accounts provide poignant glimpses into their experiences of food insecurity and accessing food banks while having to also cope with the presence of chronic health conditions such as diabetes, fibromyalgia, heart disease, osteoarthritis, depression, and anxiety.

Taken together, our findings revealed a common theme of recurring and persistent food bank access at the 18-month time point, with all participants reporting chronic use except one, who had begun to receive his pension. A comparison of the interviews from the 6-month follow-up with this one demonstrates negligible change in food insecurity and food bank access, with little to no reported improvements in health.

Our findings are consistent with previous evidence suggesting that people who rely on food banks have typically experienced prolonged poverty, low incomes, and adverse life events [16], as well as chronic health issues such as diabetes and heart disease [2–4]. In our 18-month follow-up, all eleven participants reported long-standing physical and/or mental health conditions.

As defined above, food insecurity is due to financial constraints, and income has been found to be a strong determinant of health and well-being [2, 30]. Furthermore, health problems due to hunger – an extreme outcome of food insecurity – can develop early in life, as shown by a 10-year study which found that any experiences of hunger in childhood were associated with poorer general health at the endpoint of the study, whereas repeated experiences of hunger were associated with increased risk of chronic diseases [31]. Chronic health problems can also worsen over time among people who experience food insecurity if they are not able to afford the therapeutic diets and medications that are prescribed for their conditions [32]. A large 4-year study found that severe maternal depression increased the likelihood of household food insecurity by 69% [33]. Bi-directional relationships between food insecurity and health may exist and further research is needed to elucidate them. For example, poor health could preclude obtaining or retaining well-paid employment and the cost of treatment could be financially devastating, while conversely, food insecurity could lead to stress-induced compromised health.

The onset of chronic illnesses early in life and the limited ability to self-manage these conditions due to factors related to poverty are likely significant factors in the finding that public healthcare expenses for a severely food-insecure adult in Ontario (Canada’s largest province) are, on average, more than double those of a food secure adult [2]. Tarasuk and colleagues found that, compared to food-secure households, those facing moderate food insecurity incurred 49% higher health care costs, while those who were severely food-insecure incurred 121% higher health care costs [4]. Prior research has suggested that income support programs may be linked with improved health and reduced healthcare costs. For example, a large study in Canada found significant improvements in the self-reported physical and mental health of low-income Canadians after the age of 65 when they started to receive old age pensions [34]. A study conducted in Manitoba found that the number of hospitalizations declined by 8.5% after a five-year guaranteed annual income experiment (MINCOME) [35].

According to the HungerCount 2018 report from Food Banks Canada, people who report social assistance or disability-related benefits as their main source of income account for 59% of population who rely on food banks [36]. The Food Banks Canada report also describes “a cycle of poverty that is extremely difficult to escape” among people who receive social assistance as their main source of income (p. 21), a problem which was corroborated in our original 6-month study where participants described having to live on a monthly cycle [20]. A mixed-methods study conducted in Vancouver using surveys and focus groups [37] also found that financial constraints related to insufficient income, when receiving social assistance benefits as the primary source of income, combined with rising housing costs and chronic health issues, reinforced the participants’ perception of food banks as a resource they would need to rely on over the long term.

Based on the results of this 18-month follow-up study and those of other larger studies, it is evident that the current systems of food banks cannot provide a reliable and adequate supply of food to meet the long-term nutritional needs of food-insecure Canadians. This finding highlights the need for programs that target long-term food insecurity and its underlying causes, including poverty.

### Limitations

The present study was restricted to people who accessed food banks in Ottawa, Ontario, Canada. As a result, the findings may not be generalizable to people who access food banks in other geographic regions.

Because we were only able to contact 11 of the original 29 participants from the baseline point of our previous study, the small sample size in this 18-month follow-up may limit the completeness of our findings; that is, other important themes may have emerged if more participants had been interviewed. As such, the small sample size prevented checking for data saturation (when additional interviews no longer yield substantial new information).

The high rate of attrition in itself may suggest a high degree of housing instability among people who rely on food banks, and this potentially raises further concerns for their well-being. Further research on food insecurity and housing stability may be very helpful.

## Conclusions

This study contributes contemporary evidence on the experiences of people who accessed food banks over time, through the qualitative analysis of interviews that were conducted in the 18-month follow-up. Our findings support those of previous studies, that people rely on food banks as a long-term and regular resource to try to meet their basic needs, and that these people often struggle with chronic health issues. The observed lack of change in this 18-month follow-up indicates a serious problem, and it provides further evidence that food banks are not able to alleviate chronic food insecurity.

Although the number of participants in our study is small, the longitudinal nature strives to convey the unique and personal perspectives of the participants, as they try to live their lives and carry on, despite the uncertainty that is the current state of the social safety net for the food insecure. The results of our study suggest that programs to address long-term food insecurity and its underlying causes are urgently needed to effectively mitigate food insecurity in Canada.

## Supplementary Information

**Additional file 1. **

**Additional file 2. **

## Data Availability

The datasets used and/or analysed during the current study are available from the corresponding author on reasonable request.
