# Supplementary material for: More food for thought: a follow-up qualitative study on experiences of food bank access and food insecurity in Ottawa, Canada
Source: BMC Public Health. 2022 Mar 25;22:586. doi: 10.1186/s12889-022-13015-0 (PMC8953391; doi:10.1186/s12889-022-13015-0)
Supplement: Supplementary file 1 — Additional file 1. [file 12889_2022_13015_MOESM1_ESM.pdf]

Appendix 1. Consolidated criteria for reporting qualitative studies (COREQ): completed 32-item checklist.

**Domain 1: Research team and reflexivity**	**Section – Subsection – Paragraph No.**
**Personal Characteristics**	
1. Interviewer/facilitator - Which author(s) conducted the interview or focus group?	Methods – Data Collection – Para 2
2. Credentials - What were the researcher's credentials?	Declarations – Author's Information
3. Occupation - What was their occupation at the time of the study?	Declarations – Author's Information
4. Gender - Was the researcher male or female?	Methods – Data Collection – Para 2
5. Experience and training - What experience or training did the researcher have?	Methods – Data Collection – Para 2
**Relationship with participants**	
6. Relationship established - Was a relationship established prior to study commencement?	Methods – Participants and Setting – Para 1
7. Participant knowledge of the interviewer - What did the participants know about the researcher? e.g., personal goals, reasons for doing the research?	Methods – Participants and Setting – Para 1
8. Interviewer characteristics - What characteristics were reported about the interviewer/facilitator? e.g., bias, assumptions, reasons, and interests in the research topic?	Declarations – Author's Information
**Domain 2: study design**	
**Theoretical framework**	
9. Methodological orientation and Theory - What methodological orientation was stated to underpin the study? e.g., grounded theory, discourse analysis, ethnography, phenomenology, content analysis	Methods – Data Analysis – Para 1
**Participant selection**	
10. Sampling - How were participants selected? e.g., purposive, convenience, consecutive, snowball	Methods – Participants and Setting – Para 1
11. Method of approach - How were participants approached? e.g., face-to-face, telephone, mail, email	Methods – Data Collection – Para 2

12. Sample size - How many participants were in the study?	Methods – Participants and Setting – Para 1
13. Non-participation - How many people refused to participate or dropped out? Reasons?	Methods – Participants and Setting – Para 1
**Setting**	
14. Setting of data collection - Where was the data collected? e.g., home, clinic, workplace	Methods – Data Collection – Para 2
15. Presence of non-participants - Was anyone else present besides the participants and researchers?	Methods – Data Collection – Para 2
16. Description of sample - What are the important characteristics of the sample? e.g., demographic data, date	Methods – Participants and Setting – Para 1
**Data collection**	
17. Interview guide - Were questions, prompts, guides provided by the authors? Was it pilot tested?	Methods – Data Collection – Para 1 (guide was tested in previous phase of study)
18. Repeat interviews - Were repeat interviews carried out? If yes, how many?	Methods – Participants and Setting – Para 1
19. Audio/visual recording - Did the research use audio or visual recording to collect the data?	Methods – Data Collection – Para 2
20. Field notes - Were field notes made during and/or after the interview or focus group?	n/a (phone interviews only, recorded, and transcribed verbatim)
21. Duration - What was the duration of the interviews or focus group?	Methods – Data Collection – Para 2
22. Data saturation - Was data saturation discussed?	Discussion – Limitations – Para 3
23. Transcripts returned - Were transcripts returned to participants for comment and/or correction?	n/r (not part of the methodology)
**Domain 3: analysis and findings**	
**Data analysis**	
24. Number of data coders - How many data coders coded the data?	Methods – Data Analysis – Para 1
25. Description of the coding tree - Did authors provide a description of the coding tree?	n/r (coding process is described in Methods – Data Analysis – Para 1)

26. Derivation of themes - Were themes identified in advance or derived from the data?	Methods – Data Analysis – Para 1
27. Software - What software, if applicable, was used to manage the data?	Methods – Data Analysis – Para 1
28. Participant checking - Did participants provide feedback on the findings?	n/r (not part of the methodology)
**Reporting**	
29. Quotations presented - Were participant quotations presented to illustrate the themes / findings? Was each quotation identified? e.g., participant number	Results (all subsections)
30. Data and findings consistent - Was there consistency between the data presented and the findings?	Results (all subsections, three main themes)
31. Clarity of major themes - Were major themes clearly presented in the findings?	Results (all subsections, three main themes)
32. Clarity of minor themes - Is there a description of diverse cases or discussion of minor themes?	n/a (not applicable to study objective)

n/a: not applicable; n/r: not reported
