# Supplementary material for: More food for thought: a follow-up qualitative study on experiences of food bank access and food insecurity in Ottawa, Canada
Source: BMC Public Health. 2022 Mar 25;22:586. doi: 10.1186/s12889-022-13015-0 (PMC8953391; doi:10.1186/s12889-022-13015-0)
Supplement: Supplementary file 2 — Additional file 2. [file 12889_2022_13015_MOESM2_ESM.pdf]

Appendix 2. Food Bank Client Interview Guide for 6-Month and 18-Month Follow-Ups.

Experience and interactions at the food bank:

1. Have you used the food bank in the past 6 months? If yes, how often did you go to the food bank?

2. How would you describe your overall experiences with the food bank over the past 6 months?

3. Have you noticed any changes at the food bank in the past 6 months? [changes in the food, volunteers, how it's run, people you interact with, anything at all]

Perceived diet & health:

4. In the past 6 months, have there been any changes to your diet? Have you made any changes in the foods you usually eat?

5. Have there been any recent changes in your physical health?

6. Have there been any recent changes in things that affect your mental health? Has anything changed that affects your mood, stress, happiness, or anything else related to your mental well-being?

Social support:

7. In the past 6 months, did you get involved in any new community programs? If so, could you please describe them? In what ways, if any, have these programs been helpful?

8. Have there been any other changes in your social support in the past 6 months?

9. What kinds of support, services, or programs, if any, would be most helpful for you right now?

Background and life circumstances:

1. Over the past 6 months have you experienced any major life changes? E.g., changes in where you live, your employment or income source, or anything else major?

Closing reflection:

2. Is there anything else you would like to share with us?
